# Moving and unsinkable graphene sheets immobilized enzyme for microfluidic biocatalysis

**DOI:** 10.1038/s41598-017-04216-4

**Published:** 2017-06-27

**Authors:** An Gong, Chang-Tong Zhu, Yan Xu, Fang-Qin Wang, D’assise Kinfack Tsabing, Fu-An Wu, Jun Wang

**Affiliations:** 1 School of Biotechnology & School of the Environment and Chemical Engineering, Jiangsu University of Science and Technology, Zhenjiang, 212018 P.R. China; 2 Sericulture Research Institute, Chinese Academy of Agricultural Sciences, Zhenjiang, 212018 P.R. China

## Abstract

Enzymatic catalysis in microreactors has attracted growing scientific interest because of high specific surface enabling heat and mass transfer and easier control of reaction parameters in microreactors. However, two major challenges that limit their application are fast inactivation and the inability to the biocatalysts in microchannel reactors. A fluid and unsinkable immobilized enzyme were firstly applied in a microchannel reactor for biocatalysis in this study. Functionalized forms of graphene-immobilized naringinase flowing in microchannels have yielded excellent results for isoquercitrin production. A maximum yield of 92.24 ± 3.26% was obtained after 20 min in a microchannel reactor. Ten cycles of enzymatic hydrolysis reaction were successively completed and an enzyme activity above 85.51 ± 2.76% was maintained. The kinetic parameter *V*_m_/*K*_m_ increased to 1.9-fold and reaction time was decreased to 1/3 compared with that in a batch reactor. These results indicated that the moving and unsinkable graphene sheets immobilized enzyme with a high persistent specificity and a mild catalytic characteristic enabled the repetitive use of enzyme and significant cost saving for the application of enzyme catalysis. Thus, the developed method has provided an efficient and simple approach for the productive and repeatable microfluidic biocatalysis.

## Introduction

In the past decades, microreactors have been used for many biocatalytic reactions^1^. The reactor channel has microscale geometrical features, which results in an extremely high surface-to-volume ratio providing a larger specific surface area for efficient heat and mass transfer in a short reaction time^2^. These parameters are essential for highly exothermic reactions, where the need for precise and intensive transfer of generated heat is crucial. With biocatalytic reactions in microreactors, the operating conditions (i.e. pressure, temperature, concentration, residence time, etc.) can be better controlled, which can contribute to the reduction of the by-products formation, the enhancement of the selectivity and the yield of target products. Biocatalytic reactions in microreactors have a wide range of uses, including uses in heterogeneous catalysis, homogeneous catalysis and photo catalysis. Recently, *Thermomyces lanuginosus* lipase has been used in the synthesis of natural flavor esters in a continuous flow microreactor^3^, and a crude cell lysate containing hydroxynitrile lyase has been applied to synthesize optically pure cyanohydrins in a microreactor^4^. However, in the present microreactor, these reactions may occur with the following limitations: (1) difficult separation of free enzyme from the product; (2) non-reuse of free enzyme; (3) easy inactivation of free enzyme in the microchannel.

To solve these problems, a large number of different applications involving the use of microchannel-immobilized enzyme have already been presented. Such as, immobilizing enzymes on a glass-capillary surface using a chemical modification containing sol-gel techniques has already been reported, and this process requires high-level techniques and multi-step procedures, leading to low-cost performance^5, 6^. Enzyme-immobilized microreactors have been prepared in capillaries and on microfluidic chips by immobilizing trypsin on porous polymer monoliths consisting of 2-vinyl-4, 4-dimethylazlactone, ethylene dimethacrylate, and acrylamide or 2-hydroxyethyl methacrylate^7^. For effective electronegative polymerization, an enzyme-immobilized microreactor using a cross-linking enzyme membrane on a microchannel surface has been developed as a facile and inexpensive preparation method^8^. These methods provide advantages such as the possibility to reuse the enzymatic system, lower reactant consumption, extended enzyme life time and greater stability. However, in this arrangement, the replacement of the deactivated catalyst can be technically challenging. In addition, these structures of microreactors are often responsible for the generation of high pressure, which is unfavorable for micro-synthesis systems including multiple processes. And the microfluidic biocatalysis needs a highly controlled microreactor with energy-intensive operation. Thus, a simple and inexpensive enzyme-immobilized microreactor without operation of the high pressure needs to be designed.

Recently, a vast majority of studies of enzyme immobilization have worked on nanomaterials with the potential for the target product or biocatalyst. Chitin, chitosan, activated silica and glass nanoparticles have been used as supports for enzyme immobilization^9–12^. It is worth mentioning that a core-shell structured iron oxide magnetic nanoparticle was used to immobilize *Thermomyces lanuginosus* lipase^13^. Biological reactions using immobilized enzyme can enable the easy separation of the enzyme from the product, the multiple reuses of enzyme, and a high operational stability, as well as the low cost of enzymes^14^.

Considering the extremely small size and the excellent performance in immobilizing enzymes, nanoparticles can be used as carriers of immobilized enzyme to be applied in a microchannel reactor. However, the following two important problems need to be solved firstly: (1) the non-coagulation of immobilized enzyme by nanoparticles; and (2) a high loading capacity for enzyme. Carbon-based nanomaterials, such as carbon nanotubes and graphene have attracted considerable interest among the nanostructured materials, which could provide a good opportunity to solve these problems. Especially, a larger specific surface area of nanomaterials may be conducive to higher enzyme loading and better chemical stability^15^. Functionalized carbon nanotubes have been employed for NADH oxidase immobilization^16^. Graphene, which consists of a one-atom-thick two-dimensional sheet of sp^2^-bonded carbon atoms, has been proposed as a versatile material to be applied in various fields^17–19^. Recently, graphene has been explored for the synthesis of various types of derivatives. For example, graphene sheets covalently functionalized with aromatic-aliphatic amino acids have demonstrated stable dispersion in water and in common organic solvents^20^. A new type of casein phosphopeptides and bio-functionalized graphene composite was synthesized for hydroxyapatite biomimetic mineralization by an amidation reaction^21^. In addition, surfactants and ionic liquids have successfully been used for the noncovalent surface functionalization of graphene to obtain highly stable dispersions in aqueous and organic solvents. Thus, the perfect synergy of graphene sheets, surfactants and ionic liquids maybe can solve the mentioned two problems.

**Figure 1:**
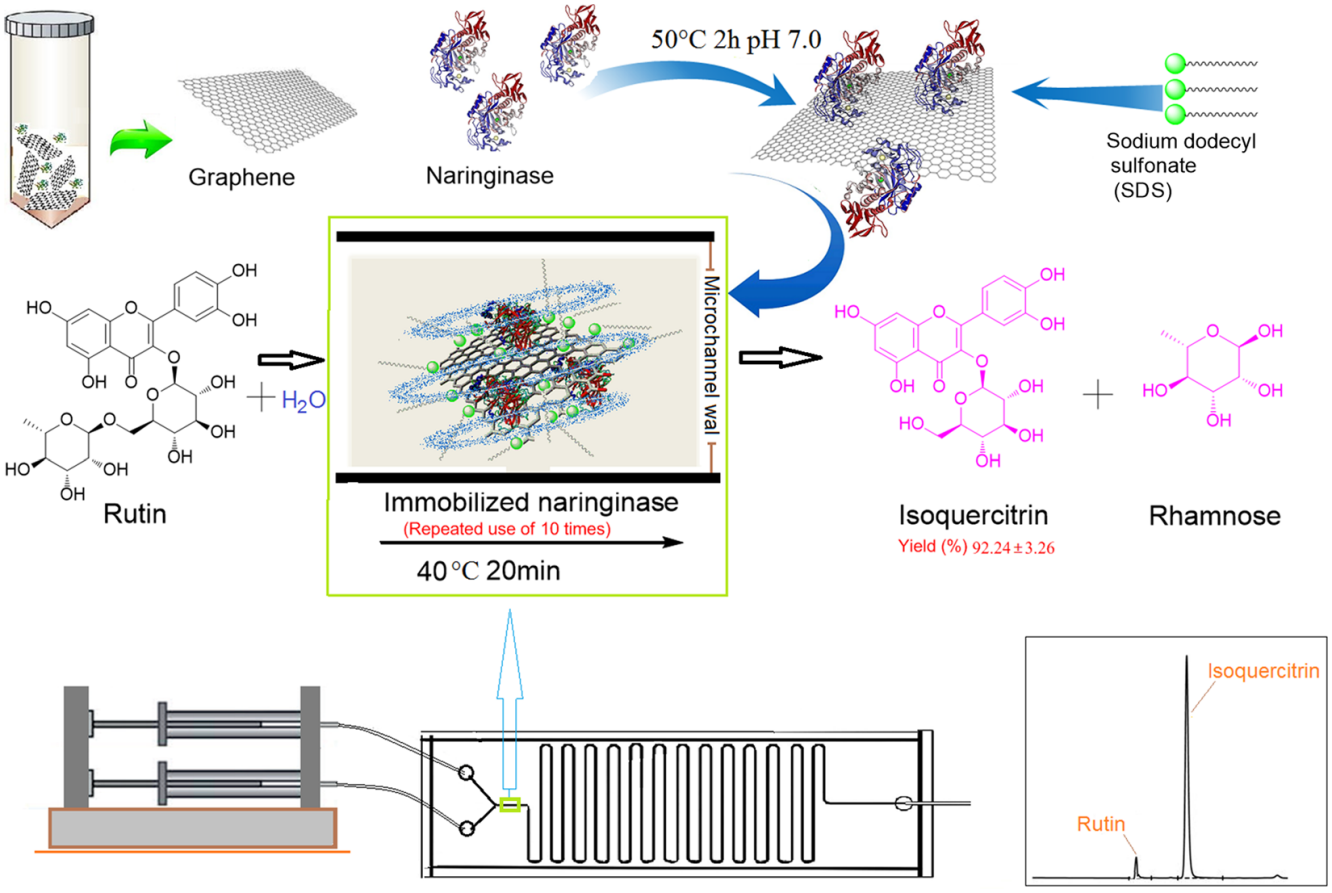
Biosynthesis diagram of isoquercitrin in a microchannel reactor with a fluid and unsinkable immobilized enzyme. The photos were taken and modified by C. Z., and the diagram was drawn by A. G.

Isoquercitrin is a rare flavonol glycoside with wide biological activities, and regards as a key intermediate for the production of enzymatically modified isoquercitrin (EMIQ)^22^. Among the reported methods, enzymatic transformation would be feasible for isoquercitrin production if the process could be performed at a reasonable cost^23^. Improving the reaction rate to achieve a high yield is the final target, so a promising enzymatic method to greatly strengthen the reaction process is urgently needed. In this study, the immobilization method used is nonspecific adsorption, which is based on physical adsorption. Immobilization by adsorption is a mild, easy-to perform process and usually preserves the catalytic activity of the enzyme. The advantages of immobilized enzyme have been combined with microreactors flexibly. A functionalized form of graphene sheets was firstly used as the immobilized carriers for biocatalysis in a micro-channel reactor (see Fig. 1).

## Results and Discussion

### Choice of nanoparticle carrier for enzyme immobilization

Table 1 shows the adsorbing capacities of different nanoparticles. Under the same condition, the adsorbing capacity was affected by nanoparticle types. The maximal adsorbing capacity of graphene nanoparticles was 1.10 g/g. On the contrary, Fe_2_O_3_ nanoparticles show the lowest adsorbing capacity with 0.42 g/g. In addition, the different groups of nanotubes nanoparticles had a similar adsorbing capacity approximately 0.92 g/g, which was a little lower than that of graphene but higher than Fe_2_O_3_ nanoparticles. Carbon nanotubes had been also used as carriers of immobilized lipase for conversion of Jatropha oil to fatty acid methyl esters, and the maximum adsorbing capacity was obtained with 0.41 g/g^24^. Thus, the specific surface area, temperature and other factors could result in the difference of the adsorbing capacity. Moreover, graphene nanoparticles had the smallest diameter (0.55–1.2 nm), length (0.5–3 *μ*m) and density (0.77 g cm^−3^). The size effect of nanoparticle particles on the adsorptive properties was obvious. As the nanoparticles size decreases, the adsorption capacity increases^25^. A similar trend was observed in the experiment on activated carbon adsorption dye in such that the same mass of graphene nanoparticles had the biggest adsorbing capacity compared to other nanoparticles^26, 27^.

**Table 1:** Different physical parameters of the immobilized supporters.

Types	Diameter (nm)	Length (*μ*m)	Density (g/cm^3^)	Adsorbing capacity ^a^ (g)
MW-CN	20–30	10–30	2.01	0.82 ± 0.03
MWC-OH	20–30	10–30	1.96	0.99 ± 0.02
MWC-COOH	20–30	10–30	2.12	0.95 ± 0.05
SMW	20–30	0.5–2	1.68	0.89 ± 0.06
SMW-OH	20–30	0.5–2	1.62	0.86 ± 0.14
SMW-COOH	20–30	0.5–2	1.73	0.89 ± 0.04
MW-Ni	20–30	10–30	1.98	1.02 ± 0.13
Fe_2_O_3_	—	30–50	5.18	0.42 ± 0.07
Graphene	0.55–1.2	0.5–3	0.77	1.10 ± 1.81

a: Reaction condition: the temperature was 50 °C; the enzyme powder was confected in 20 g/L by 2 mL disodium hydrogen phosphate-citrate buffer (pH 7); the nanoparticles mass was 10 mg; the reaction time was 3 h; the mixture was stirred at 120 rpm in an incubator shaker.

The nanoparticles-immobilized enzyme would be used in the microchannel reactors, so the size and density of the carriers needs to be of an appropriate range. When the size of the nanoparticle was larger than that of the channel, it might lead to the blockage of the microchannels. Moreover, the density of nanoparticles was also an important factor. The larger density of nanoparticles could deposit onto the bottom, while they flowed in the microchannels. Considering the overall factors, graphene were found to be the most suitable nanoparticles for use as carriers and could be applied in a microchannel reactor.

### Characterization of immobilized enzyme

**Figure 2:**
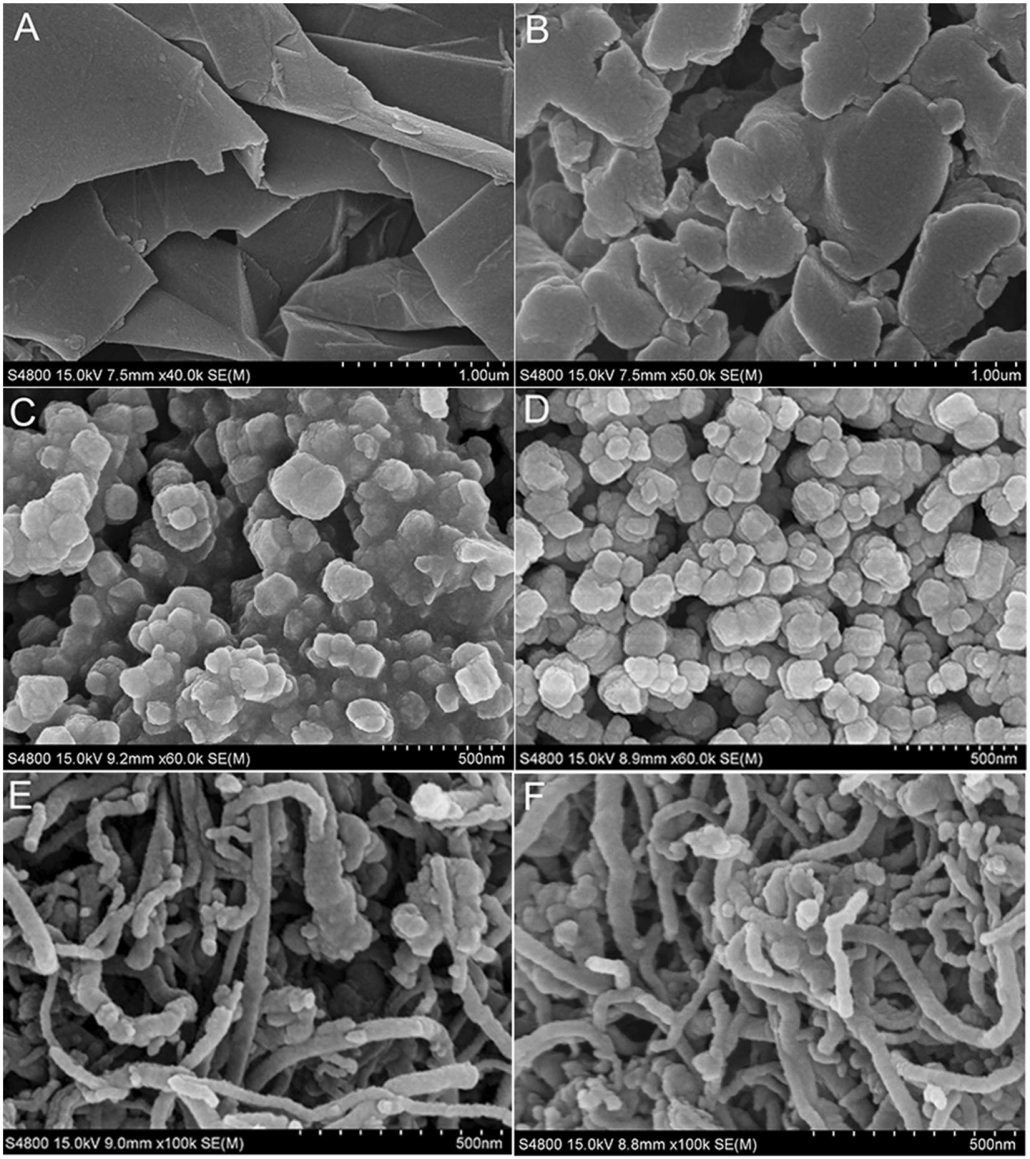
The SEM photos of pure graphene (**A** and **B**), Fe_2_O_3_ (**C** and **D**) and carbon nanotube nanoparticle (**E** and **F**) before and after immobilizing. Reaction condition: enzyme solution (20 g/L) was dissolved by disodium hydrogen phosphate-citrate buffer (pH 7); graphene nanoparticles mass (10 mg) was added in 2 mL of enzyme solution and the mixture was stirred at 120 rpm in an incubator shaker for 3 h, reaction temperatures was 50 °C.

Figure 2A depicts the image of graphene before immobilizing procedure. After the immobilization, it was observed that graphene sheets were evenly attached by naringinase (Fig. 2B). Figure 2C and D shows the differences on the surface micromorphology of Fe_2_O_3_ nanoparticles before and after immobilizing were not significant. It indicated that a small quantity of naringinase had attached to Fe_2_O_3_ nanoparticles. For carbon nanotubes, a few morphological changes were observed and shown in Fig. 2E and F. Their SEM images revealed the excellent performance of graphene used as carriers for naringinase immobilization.

**Figure 3:**
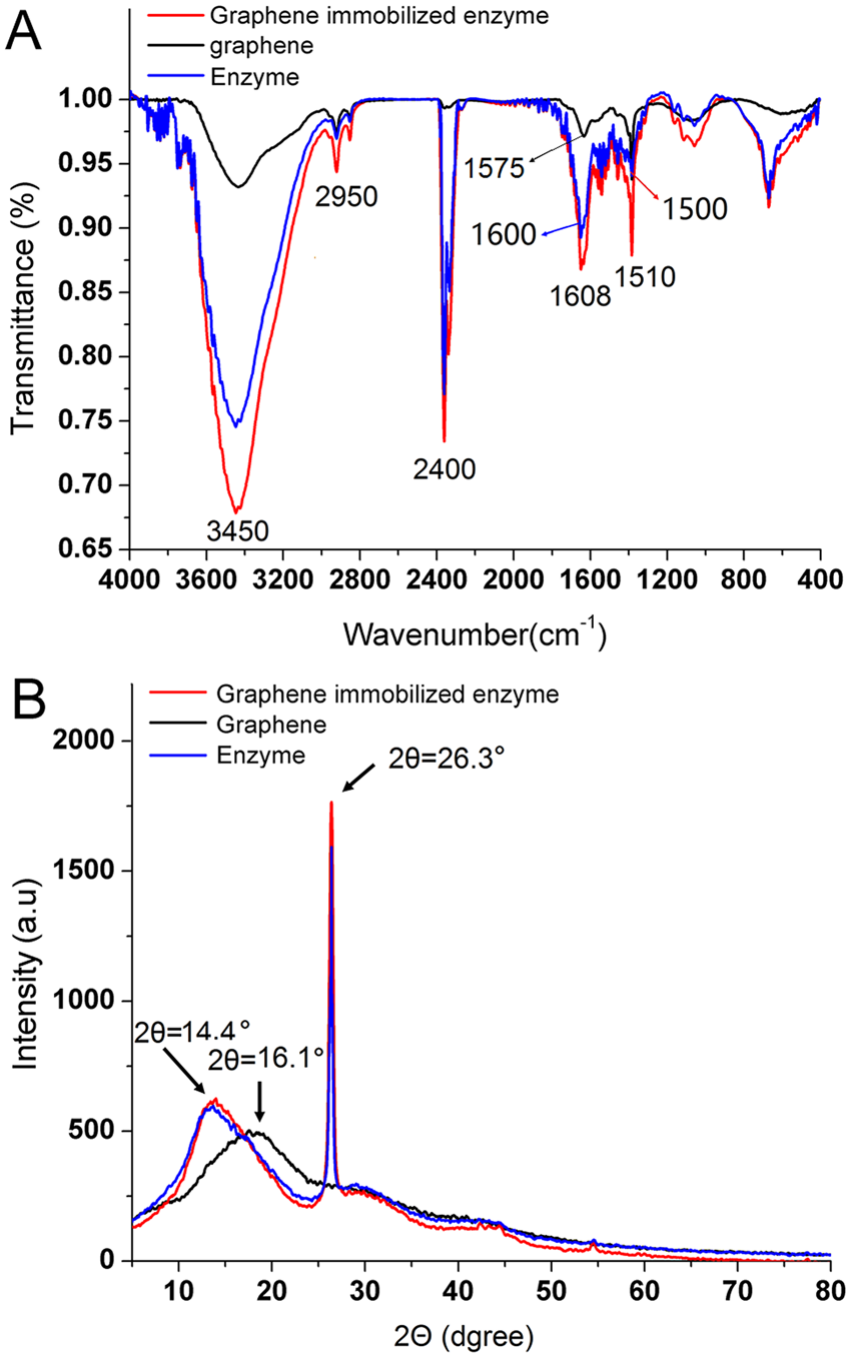
The FT-IR (**A**) and XRD (**B**) spectra of graphene immobilized enzyme, graphene and enzyme. Reaction condition: enzyme solution (20 g/L) was dissolved by disodium hydrogen phosphate-citrate buffer (pH 7); graphene nanoparticles mass (20 mg) was added in 4 mL of enzyme solution and the mixture was stirred at 120 rpm in an incubator shaker for 3 h, reaction temperatures was 50 °C.

Figure 3A shows the FT-IR spectra of graphene, naringinase and graphene-immobilized naringinase, which displayed the changes in absorption peaks. The emission spectrum of graphene sheets showed peaks at 3450 cm^−1^, 2950 cm^−1^, 2400 cm^−1^, 1500 cm^−1^ and 1575 cm^−1^. The absorption peaks at 1500 cm^−1^ and 1575 cm^−1^ assigned to the aromatic C=C stretching vibration both displayed increased intensity, and red shifts in the peaks from 1500 cm^−1^ to 1510 cm^−1^, and from 1575 cm^−1^ to 1608 cm^−1^, respectively. The absorption band at about 3450 cm^−1^ could be attributed to the stretching vibration of hydrogen-bonded hydroxyl groups. It should be noted that there was a significant change in the band intensities at 3450 cm^−1^ after the immobilization due to abundant hydroxyl groups. The result confirmed that the hydroxyl groups from naringinase were successfully connected with graphene sheets. Moreover, the peaks at about 1608 cm^–1^ was caused by the stretching vibration of C=O bond due to amide groups from naringinase. Meanwhile, a significant absorption peak was found in the FT-IR spectra of pure enzyme at 1600 cm^−1^. This property further confirmed the enzyme molecules were adsorbed by graphene sheets. A different result was shown in the FT-IR spectra when *cicer α*-galactosidase was immobilized on graphene^28^. Compared with this reference, the light transmission of graphene-immobilized naringinase was lower. It suggested that more naringinase molecules were immobilized on the graphene. Due to the different adsorption capacity of graphene for different enzyme molecules, and the results observed, naringinase could be much more easily adsorbed by graphene.

As shown in Fig. 3B, X-ray diffraction (XRD) was applied to acquire detailed information about the microstructure of graphene-immobilized naringinase. The broad diffraction peak at 2θ = 16.1° observed in amorphous regions was attributed to graphene sheets. The XRD pattern of the prepared immobilized naringinase displayed two peaks at 2θ = 14.4° and 2θ = 26.3°, respectively. The significant change of peak intensity was exhibited at 2θ = 14.4° after the immobilization. The diffraction peak at 2θ = 26.3° was narrow and the intensity was obviously increased, implying that the crystalline sizes of graphene particles were expanded after the immobilization. The changes in peak intensity might be caused by the interaction between graphene surface and enzyme protein, which changed the graphene structure. Moreover, the crystal structure of Form Ι was displayed by the peak of 2θ = 16.1° before the immobilization; However, a new crystal structure of Form IΙ was appeared by a narrow peak of 2θ = 26.3° after the immobilization, which implied an excellent crystallization had been taken place. The higher narrow peak intensity after the immobilization indicated that more enzymes were immobilized on the surface of the carriers, demonstrating a better performance of the immobilization. For instance, in the study of lipase immobilization, the narrow peak intensity was low and the maximum protein loading was only 0.48 g/g of support^29^. However, a higher narrow peak intensity and a maximum of protein loading (1.30 g/g of support) were observed after the naringinase immobilized on graphene. Thus, the calculated values of the maximum protein loading were well matched with their peak intensity in the spectra.

### Adsorption isotherm and kinetics models

**Figure 4:**
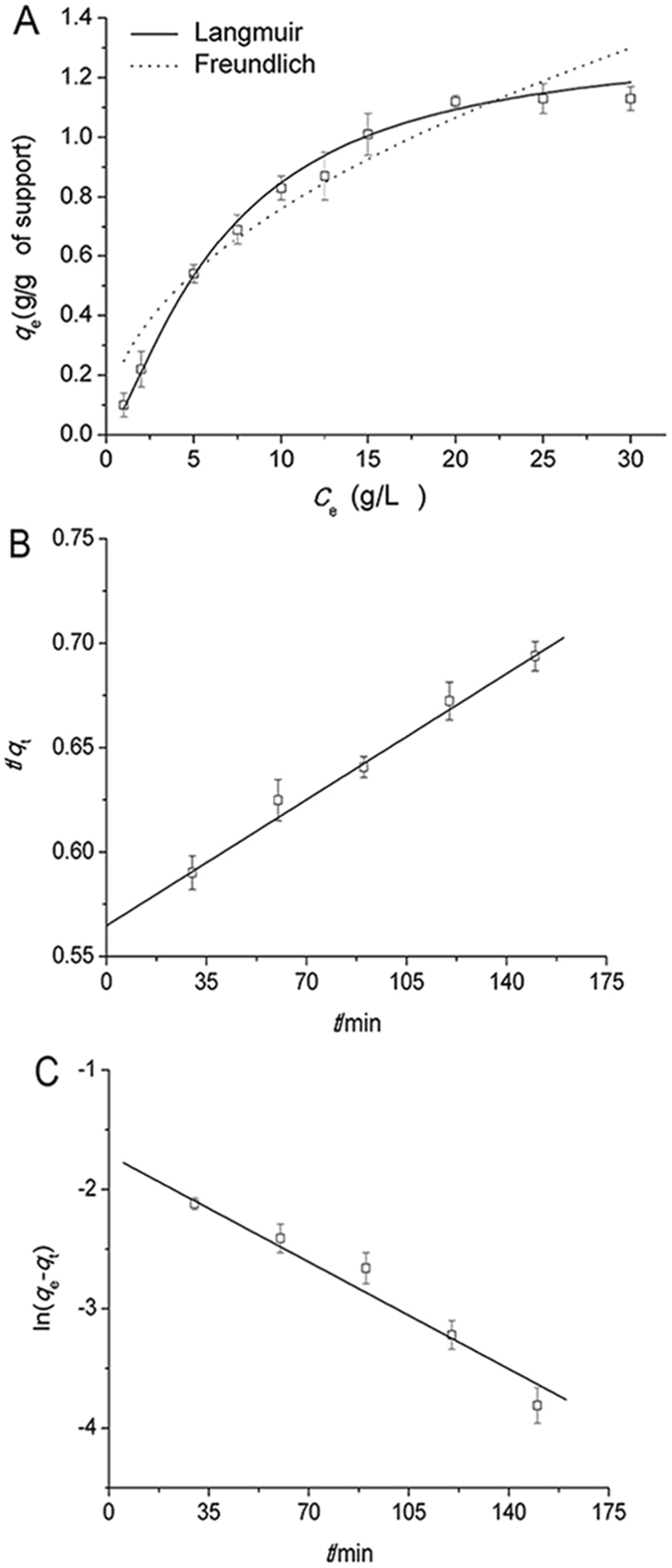
Langmuir and Freundlich isotherms (**A**), pseudo-second-order (**B**) and pseudo-first-order (**C**) adsorption kinetics of enzyme adsorped on graphene. Reaction condition: enzyme solution (20 g/L) was dissolved by disodium hydrogen phosphate-citrate buffer (pH 7); graphene nanoparticles mass (20 mg) was added in 4 mL of enzyme solution and the mixture was stirred at 120 rpm in an incubator shaker for 3 h, reaction temperatures was 50 °C.

Figure 4A shows the equilibrium adsorption behavior of naringinase immobilized on graphene. To describe how adsorbed molecules interact with the adsorbent surface, two adsorption isotherm models of Langmuir and Freundlich were performed by fitting. Both models have been the most commonly used to explain the interaction between enzyme molecules and support surfaces and to predict their equilibrium parameters. In the Freundlich model, the calculated value of *n* for naringinase adsorption onto graphene was 2.5, which in the range 2–10. Thus, the result suggests there was a good adsorption of naringinase on the graphene sheets. For the two isotherms studied, the best-fit equilibrium model was determined based on the correlation coefficient R^2^ using non-linear regression methods. The fitted curve using the Langmuir model showed a correlation coefficient greater than 0.99, with is higher than that of the fitted curve using the Freundlich model with an R^2^ value of 0.96. A similar result was found in the experiment of dye adsorbed on graphene^30^. This result suggested that the enzyme molecules were adsorbed on the surface of graphene sheets with a monolayer style. It was because that the Langmuir isotherm model is based on the assumption of monolayer adsorption on the support surface containing a finite number of sites of uniform energies of adsorption^31, 32^. Under certain conditions, a dynamic balance was established between adsorption and desorption. However, the Freundlich adsorption equation is difficult to distinguish the presence of monolayer or multilayer. This determination of the Freundlich adsorption is based on coverage and is related to the solution concentration and temperature^33^. Thus, the Langmuir isotherms were selected as the suitable fitting models for enzyme adsorbed by graphene.

As shown in Table 2, the experimental value of *q*_e_ was 1.15 g/g of support. Compared to soybean peroxidases adsorbed onto commercial single-walled carbon nanotubes with a maximum loading of 0.575 g/g^34^, graphene showed much better adsorption capability. The hydrolytic activity of the enzymatic preparation increased from 40.12 to 270.17 IU/g of support when the initial protein loading was increased from 0.2 to 5 g/g of support. When the immobilization performed was conducted on 4 g/g of support or more, no significant increase was observed in the hydrolytic activity. An increase in initial protein loading by 25-fold (from 0.2 to 5 g/g of support) resulted in an increase in hydrolytic activity by approximately 6.7-fold.

**Table 2:** Influence of initial graphene protein loading on the catalytic properties of immobilized particle.

Enzyme loading (g/g of support)	Immobilization yield (%)	Immobilized protein loading (g/g of support)	Hydrolytic activity (U/g of biocatalyst)	Specific activity (U/g_IP_)
0.2	52.19 ± 1.32	0.10 ± 0.02	40.12 ± 1.23	401.05 ± 3.51
0.4	65.69 ± 2.36	0.26 ± 0.08	78.45 ± 3.28	301.56 ± 2.45
1	28.09 ± 0.10	0.28 ± 0.06	92.78 ± 2.11	331.15 ± 1.87
1.5	22.00 ± 1.35	0.33 ± 0.01	101.36 ± 3.82	307.02 ± 3.88
2	18.98 ± 0.12	0.37 ± 0.02	124.07 ± 1.76	335.14 ± 2.65
2.5	18.61 ± 0.56	0.47 ± 0.01	144.81 ± 2.26	308.18 ± 1.45
3	23.05 ± 2.83	0.69 ± 0.03	171.93 ± 2.55	249.15 ± 3.93
4	28.25 ± 1.28	1.13 ± 0.13	265.45 ± 1.12	234.93 ± 1.23
5	23.00 ± 0.21	1.15 ± 0.21	270.17 ± 2.64	234.89 ± 2.46

Reaction condition: Different concentrations of enzyme solution (1, 2, 5, 7.5, 10, 12.5, 15 and 20 g/L) was dissolved by 2 mL disodium hydrogen phosphate-citrate buffer (pH 7); and reaction temperature was 50 °C; the nanoparticles mass was 10 mg; the mixture was stirred at 120 rpm in an incubator shaker for 3 h.

Based on the two adsorption kinetics models, curve fitting was performed and the resulting kinetics plots are shown in Fig. 4B and C. The R^2^ values for pseudo-first-order and pseudo-second-order equations were 0.93 and 0.98 respectively, which indicated a better fit to pseudo-second-order rate model than pseudo-first-order rate model. The theoretical value of *q*_e_ for the pseudo-second-order was 1.17 g/g, while the value for the pseudo-first-order was 0.99 g/g. However, the experimental *q*_e_ value was 1.15 g/g of support. The difference between experimental and theoretical values could be attributed to the adsorption of other stabilizing compounds and/or impurities present in solutions on the support surface. The result indicated that the adsorption of enzymes onto graphene followed by the pseudo-second-order rate model, which is better than that by the pseudo-first-order model. Moreover, the kinetic behavior of lead ions adsorbed by magnetic chitosan/graphene oxide has similar results^35^. It was because the two carriers, graphene and graphene oxide, have similar structure layer characteristics.

### Functionalized forms of graphene-immobilized naringinase for flowing in microchannels

**Figure 5:**
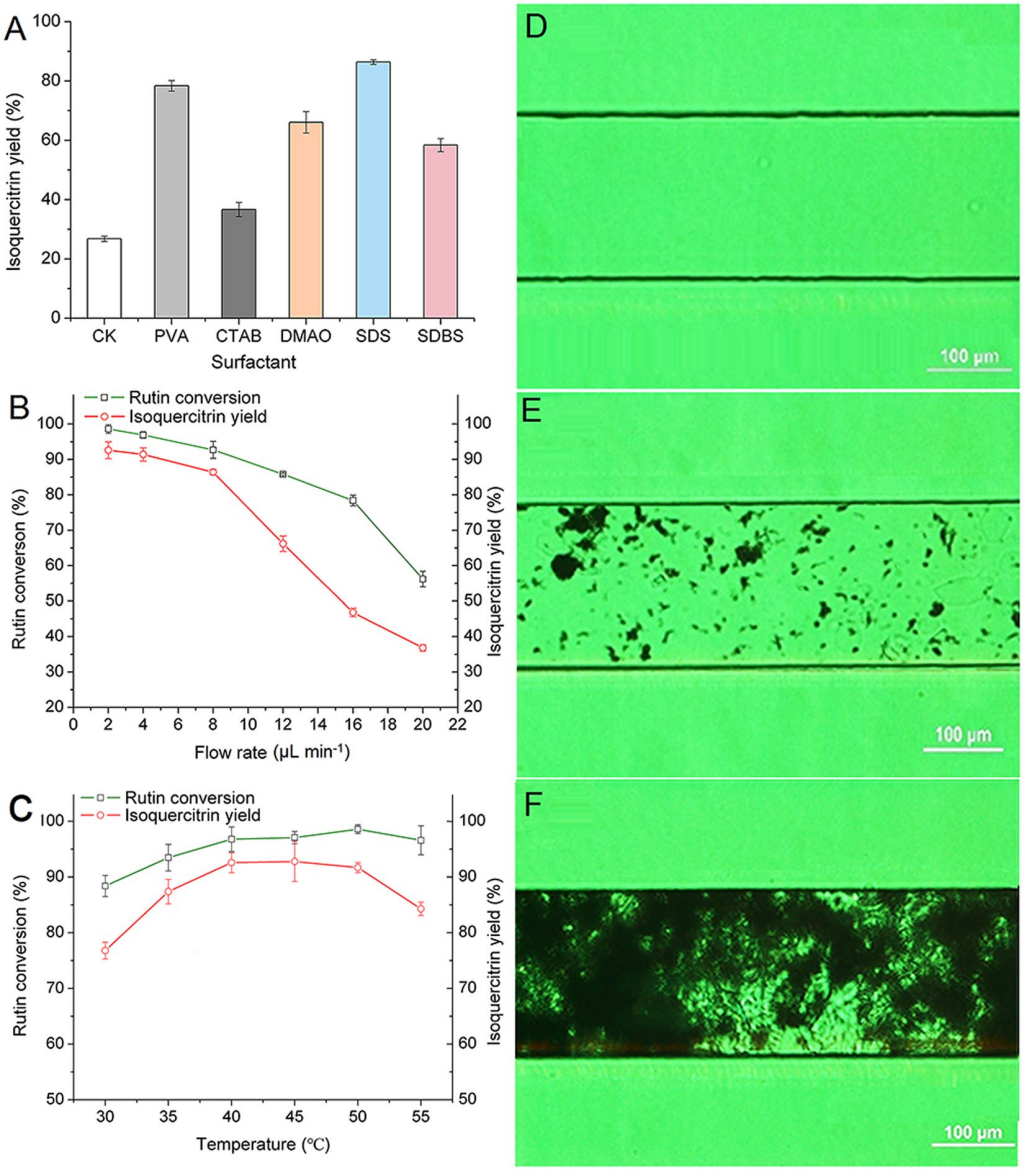
Effect of various reaction parameters on isoquercitrin yield in the microchannel reactor, including different surfactants (**A**), temperature (**B**) and flow rate (**C**). Reaction conditions: rutin concentration was 0.05 g/L. The real-time microchannel graph under inverted fluorescence microscope when inserting the reaction system in different stages; (**D**) before injecting graphene immobilized enzyme; (**E**) after injecting graphene immobilized enzyme for a moment; (**F**) stabilization after injecting graphene immobilized enzyme.

Figure 5A shows graphene is functionalized using five different surfactants for better application in a microchannel reactor. Figure S1 depicts that graphene can stably disperse in water with SDS, PVA and SDBS. A similar result was also applied on solubilizing high weight fraction single-wall carbon nanotubes in water by the nonspecific physical adsorption of sodium dodecyl benzene sulfonate^36^. Graphene functionalized by sodium dodecyl sulfonate (1 g/L) possessed a better dispersion, hydrophilia and catalytic activity in the aqueous solution. By adding the sodium dodecyl sulfonate to the reaction system, the yield of isoquercitrin increased to 87.52% while it only reached 26.86% yield without any surfactant. This result occurred because alkyl sulfonic acid salts surfactant has the ability to better reduce the interfacial tension of water, which cause graphene-immobilized enzyme to flow more smoothly and to become more suspended in the reaction system. Under the state of suspension, graphene-immobilized enzymes were allowed to gain contact with substrates more completely. Functionalized forms of graphene-immobilized enzyme have presented a good performance for raising the yield in the microchannel reactor, relatively.

The morphology of the immobilized enzyme flowing in the microchannel reactor was studied using fluorescence inversion microscope. Before injecting graphene-immobilized enzyme, the microchannel shown in Fig. 5D was empty. After the graphene-immobilized enzyme was injected, Fig. 5E depicts a small amount of immobilized enzyme flowing into the microchannel. In addition, Fig. 5F shows the high concentrations and uniform dispersion of the immobilized enzyme formed in the microchannel reactor after the injected graphene-immobilized enzyme reaching to a steady state. By considering previous studies with these results simultaneously, the final formation of flowing immobilized enzyme in the microchannel occurred due to the smaller density of graphene and the absence of surfactant in the reaction system. With better dispersion in the microchannel, more active sites of the immobilized enzyme with the final formation were exposed to the substrate molecule which increased the chances for the combination of substrate and enzyme. The results also indicated that flow rate was a vital factor to microfluidic biocatalysis, because a proper flow rate could provide a sufficient residence time for the immobilized enzyme flowing in the microchannel. Moreover, more enzyme collisions were conducted and the heat transfer was exchanged with the external environment in the microchannels.

Figure 5B shows that different yields of isoquercitrin were obtained at various flow rates. Flow rates of 2* μ*L/min and 4 *μ*L/min resulted in a higher yield than the other rates, reaching 92.14% and 91.23%, respectively. As the flow rates further increased to 8* µ*L/min, the yield was reduced. In a fixed microchannel reactor, flow rate determines the residence time, and upon reducing the flow rate, the residence time increases. With longer residence times, a more complete, full contact between substrates and enzymes was allowed^37, 38^. When the flow rates were 2 *μ*L/min and 4 *μ*L/min, the residence times were 40 min and 20 min, respectively. A higher yield was achieved at the flow rate of 2 *μ*L/min. In a fixed microchannel reactor, flow rate determines the residence time, and the reaction capacity. Thus, the space-time yield depends on flow rate and the yield. Considering the space-time yield, 4 *μ*L/min was selected as the suitable flow rate for further study in the microchannel reactor.

Figure 5C shows the effects of temperature on the yield in a continuous-flow microreactor. Temperature can affect the flow pattern, the heat transfer and mass transfer in a microchannel, especially for the reaction rate and the final product yield directly. With the advantages of the microreactor, the yield was higher than that in a batch reactor. In particular, a significantly higher yield catalyzed by graphene-immobilized was observed at a lower temperature of 30 °C. At a temperature of 40 °C, the highest yield of 92.24% was achieved. Upon further increase in the temperature, ranging from 40 °C to 50 °C, the yield showed no significant increase. However, the yield started to show decline at 55 °C, because higher temperatures have been demonstrated to lead to a decline in yield and enzyme denaturation, thus lowing the efficiency of enzymatic hydrolysis. Hence, in a continuous-flow microreactor, temperature played a crucial role in the isoquercitrin production, and 40 °C was selected as the suitable temperature for the enzymatic reaction.

These results suggested that heat transfer was compromised in a microchannel and that local temperature changes were significant^39^. Forced convection heat transfer was formed to enhance heat transfer and mass transfer since the fluid injected by a syringe pump into the microreactor via Y-shaped inlets. The intensive and efficient heat transfer, enhanced in a microchannel, could lead to a shorter reaction time. However, this transfer might also result in enzyme inactivation at high temperatures. The inactivation usually is due to heat dissipating faster at the enzyme active site, which subsequently lowers the yield when the temperature is higher than the optimum value.

### Kinetic parameters of graphene-immobilized enzyme

**Figure 6:**
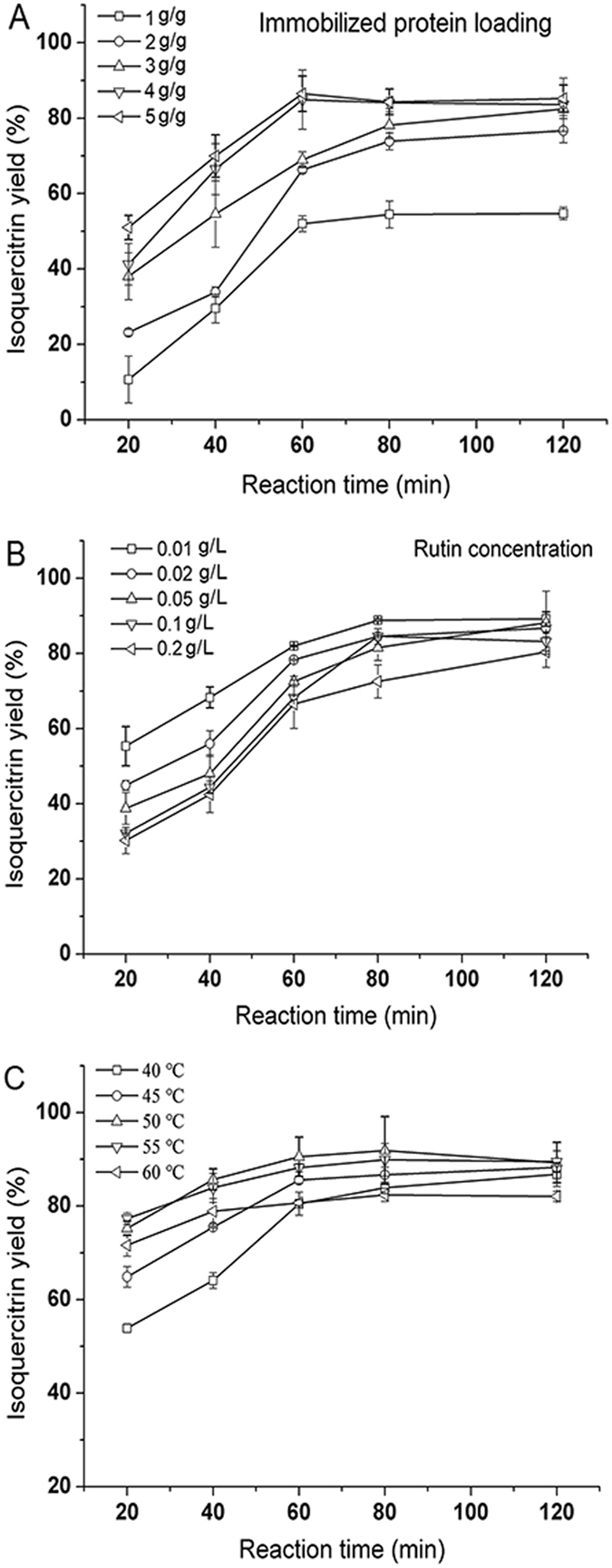
Effect of immobilized protein loading (**A**), rutin concentration (**B**) and reaction temperature (**C**) on isoquercitrin yield in a batch reactor. Reaction conditions: the mixture was in a 4 mL reaction system and obtained at 20, 40, 60, 80 and 120 min. (**A**) rutin concentration was 0.1 g/L and the mixture was shaken at 50 °C; (**B**) the nanoparticle mass was 20 mg (protein loading of 4 g g^−1^ of support) and the mixture was shaken at 50 °C; (**C**) the rutin concentration was 0.1 g/L and the nanoparticle mass was 20 mg (protein loading of 4 g/g of support).

Figure 6A shows the effect of immobilized protein loading on the yield of isoquercitrin. The concentration of the enzyme in reaction system depends on the amount of graphene-immobilized enzyme. The yield of isoquercitrin was only 11.58% after 40 min when the protein loading reached 1 g/g of support. Upon a further increase of nanoparticle mass, the yields initially increased. It was found that the protein loading increased to 4 g/g of support, the yield reached 86.57% after 1 h reaction, which was much higher than before. By the protein loading increased to 5 g/g of support, no significant increase in the ultimate yields was observed, even though the initial yields were higher. Hence, the final optimized protein loading obtained was 4 g/g of support.

Figure 6B shows the effect of rutin concentration on the yield. When the concentration of rutin was 0.01 g/L, the highest yield of approximately 89.12% was achieved in 1.33 h. Further increase in the rutin concentration decreased the yield initially compared to that initially observed. After 1.33 h, with the stepwise addition of rutin concentration, the yield increased significantly. In particular, for higher rutin concentrations (0.1 g/L), the yield reached 86.36%. However, when the rutin concentration increased from 0.1 to 0.2 g/L, the yield decreased. The probable reasons for those results include the following two factors: (1) the naringinase concentration in this experiment was very limited, resulting in an enzyme that could not fully come in contact with the substrate; (2) substrate inhibition occurred, and the enzyme was denatured, which was detected at a high substrate concentration^40^. In a batch reactor, the residence time and the reaction capacity are constant. Thus, the mass and the space-time yield both depend on the substrate concentration and the yield. Considering the space-time yield, 0.1 g/L was selected as the optimum concentration of rutin for further study.

Figure 6C shows the effect of reaction temperature on the yield. When the temperature reached 40 °C, the yield was only 52.5% at 20 min and the reaction rate was the lowest. The yield initially increased with temperatures ranging from 40 °C to 50 °C. The yield could reach 90.46% at 50 °C within 1.33 h. Above 50 °C, a decreased yield was observed, because higher temperatures led to a decline in the yield and enzyme denaturation, which lowered the efficiency of enzymatic hydrolysis. The relative stability of graphene-immobilized naringinase at the 50 °C was 25% higher than that of the free enzyme^41^. It showed the thermal inactivation at increasing temperatures was lower, compared with the free enzyme. Hence, 50 °C was selected as the optimum temperature.

**Figure 7:**
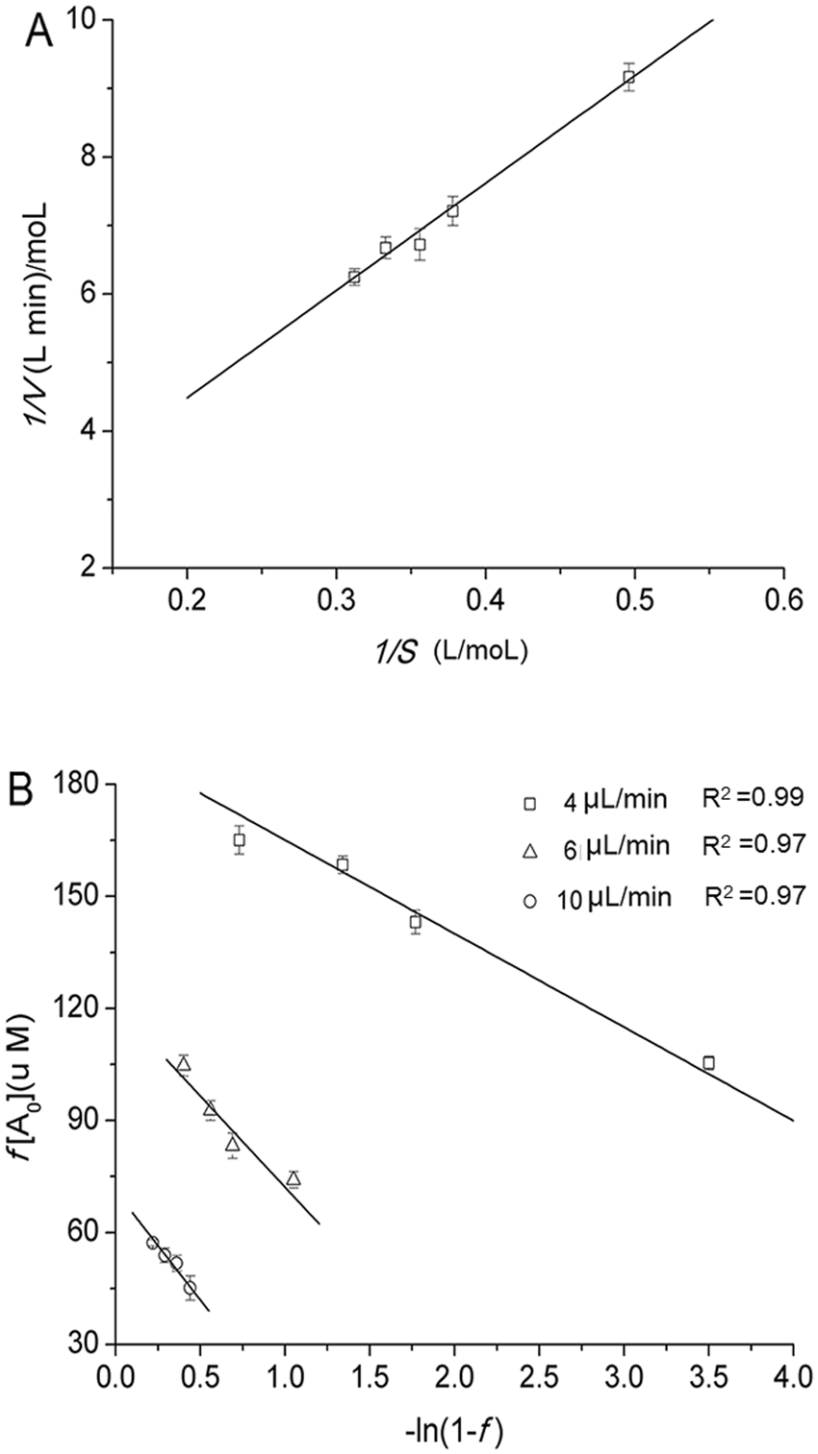
Lineweaver-Burk double reciprocal plots (**A**) and Lilly-Hornby plots (**B**) for graphene immobilized enzyme assay in a batch reactor and a microchannel reactor. Reaction conditions: (**A**) the rutin concentration was 0.1 g/L, the nanoparticle content was 20 mg, the reaction time was 1 h, and the reaction temperature was 50 °C; (**B**) the flow rates in microchannel reactor were 2 *μ*L/min, 5 *μ*L/min, and 10 *μ*L/min; rutin concentrations ranged from 0.18 to 0.32 mM.

The best optimized conditions for enzymatic synthesis of isoquercitrin were used to study studying kinetic parameters in the different reactors. Figure 7A and B illustrate the Lineweaver-Burk double reciprocal plot and Lilly-Hornby model plot for the kinetic parameters estimated for the immobilized naringinase in different reactors. Figure 7B shows the linear plots of *f* [*A*_*0*_] versus ln (1–*f* ), and *K*_m(app)_ values which were derived from the slopes of these plots. The *K*_m(app)_ values calculated by linear fitting for different flow rates in the microreactor increased in the following order: 10 *μ*L/min (48.85 *μ*M/min, R^2 = ^0.97) > 6 *μ*L/min (31.02 *μ*M/min, R^2 = ^0.98) > 4 *μ*L/min (25.17 *μ*M/min, R^2 = ^0.99). The *K*_m(app)_ values increased with increasing flow rate. The different *K*_m(app)_ values suggested that the apparent kinetics of the microreactor were significantly affected by the mass transfer. The lowest *K*_m(app)_ value was observed at a flow rate of 4* μ*L/min, indicating a more efficient interaction between enzyme and substrate. Therefore, the low flow rate is more suitable for the reaction.

As shown in Table 3, under the optimum conditions, the best results were produced in a microchannel reactor, with a *K*_m_ of 25.17 *μ*M and a *V*_m_ of 4.86 *μ*M/min. Similar results were reported by Amaro *et al* ^42^. The kinetic parameters in a microreactor were higher than that in a batch reactor and the *V*_m_ of 4.86 *μ*M/min was much higher than *V*_m_ of 3.23 *μ*M/min obtained using the free enzyme in a microreactor^43^. In addition, the kinetic parameters in a batch reactor were obtained, comprising a *K*_m_ of 36.04 *μ*M and a *V*_m_ of 3.62 *μ*M/min. From these calculated data, the value of *V*_m_/*K*_m_ when reacting in a microreactor was higher than that in a batch reactor with graphene immobilized enzymes, which indicated that substrates had a higher affinity to immobilized enzyme in a microreactor. The results indicated the consequence of efficient reaction-diffusion dynamics in the microchannel system^44^, where graphene immobilized enzymes were provided more opportunities for contacting with substrate molecules in a microreactor.

**Table 3:** Comparative results for the enzymatic-catalyzed transformation of rutin to produce isoquercitrin in different reactors.

Reactor type	Rutin concentration (g/L)	Temperature (°C)	Reaction time (min)	Isoquercitrin yield (%)	Isoquercitrin/enzyme (g/g)
Batch reactor ^a^	0.1	50	60	90.47 ± 2.89	1.74
Microreactor ^b^	0.05	40	20	92.24 ± 3.26	1.58

a: Reaction condition: rutin concentration 0.1 g/L, reaction temperature 50 °C, 120 r/min in an incubator shaker, reaction time 1 h, graphene immobilized naringinase was suspended in a reaction system;

b: Reaction condition: rutin concentration 0.05 g/L, reaction temperature 40 °C, flow rate 4 *μ*L/min, reaction time 20 min, functionalized form of graphene immobilized naringinase flowed into a microchannel reaction system.

### Reusability and relative enzyme activity of graphene-immobilized enzyme

**Figure 8:**
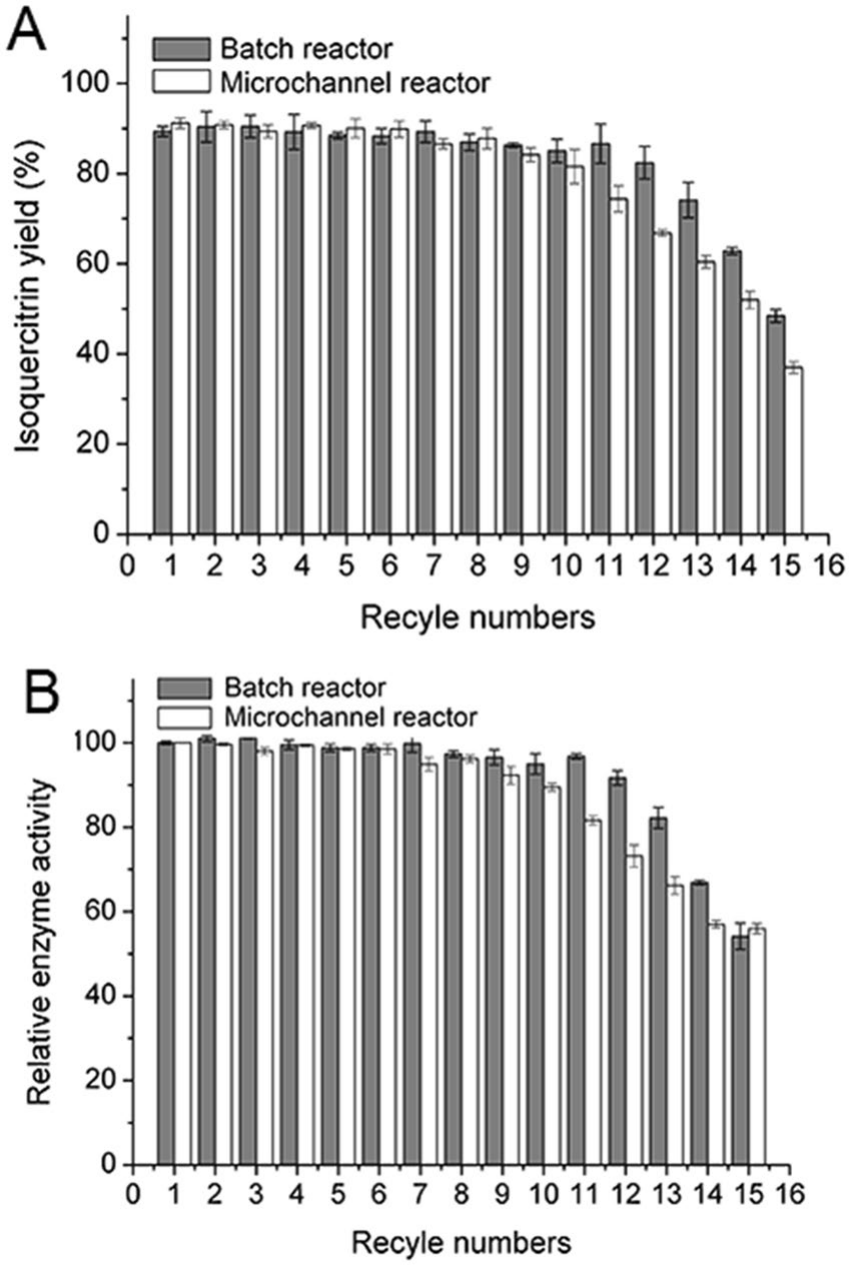
Reusability (**A**) and relative enzyme activity (**B**) of immobilized enzyme in a batch reactor and a microchannel reactor. Reaction conditions: In a batch reactor, the rutin concentration was 0.1 g/L, the nanoparticle content was 20 mg, the reaction time was 1 h, and the reaction temperature was 50 °C. In a microchannel reactor, the rutin concentration was 0.05 g/L, the reaction time was 20 min, the flow rate was 4 *μ*L/min, and the reaction temperature was 40 °C.

Figure 8A shows that under the optimum operating conditions, the graphene-immobilized enzymes were reused twelve times in a batch reactor with steady yields (80%), which demonstrated a high catalytic activity and stability. A similar result was also observed in the synthesis of galacto-oligosaccharides by *Aspergillus oryzae β*-galactosidase immobilized on magnetic polysiloxane-polyvinyl alcohol which was reutilized 10 times and retained approximately 84.33% of the initial activity^45^. The decrease of enzyme activity in a batch reactor could be due to the leakage of the enzyme during the washing process^46^. To some extent, using the non-covalent technique to immobilizing enzyme easily led to the inactivation of the enzyme^47^. On the other hand, the life of the enzyme was decreased gradually with the reutilization for several recycles.

After twelve cycles of reaction, decreased yields were observed in Fig. 8A. Figure 8B shows the decrease of the relative enzymatic activity of graphene-immobilized enzymes when compared with first reusing of the immobilized enzyme. In a microchannel reactor, ten cycles of the reaction showed high yields of isoquercitrin (above 80%) and the graphene immobilized enzymes maintained a relative enzymatic activity above 85.51%. The number of reused times was less than that in a batch reactor, which could be attributed to the stronger interaction of the immobilized enzyme with the support microenvironment in a microchannel. Sometimes, the stronger interaction resulted in enzymes detachment from carriers of graphene nanoparticles^48^.

The results suggested operational stability could be increased with enzymes immobilized by graphene in batch reactors or microchannel reactors. Meanwhile, the immobilized enzymes could also maintain its mild characteristic compared with free enzymes. However, in comparing results for the isoquercitrin production in different reactors, it was found that lower reaction temperature (reduced by 10 °C) and fewer reaction times (decreased by 50%) were required in microchannel reactors. Table 4 shows the production of isoquercitrin produced with a unit quantity of enzyme was 1.74 g/g and 1.45 g/g in different reactors, respectively. The different values indicated that more efficient enzymes utilization for obtaining higher yields occurs in microchannel reactors, even though a small amount of enzyme activity was lost due to the fast heat and mass transfer. Enzymes immobilized on graphene also enabled the repetitive use of enzymes and significant cost savings for the application of enzyme catalysis.

**Table 4:** Kinetic constants of free and graphene immobilized naringinase in different reactors.

Reactor type	Enzyme	*K*_m_ (*μ*M)	*V*_m_ (*μ*M/min)	*V*_m_/*K*_m_ (min^−1^)	Ref.
Microchannel reactor ^a^	free	—	3.23	—	Wang *et al*.^43^
Batch reactor ^b^	Immobilized	36.04	3.62	0.10	This work
Microchannel reactor ^c^	Immobilized	25.17	4.86	0.19	This work

a: Reaction condition: rutin concentration 1 g/L, reaction temperature 40 °C, flow rate 2 *μ*L/min for 40 min, [Bmim][BF_4_]-buffer (pH 9.0) (10/90, v/v) as the reaction medium;

b: Reaction condition: rutin concentration 0.1 g/L, reaction temperature 50 °C, 120 r/min in an incubator shaker, reaction time 1 h, graphene immobilized naringinase was suspended in a reaction system;

c: Reaction condition: rutin concentration 0.05 g/L, reaction temperature 40 °C, flow rate 4 *μ*L/min, reaction time 20 min, functionalization form of graphene immobilized naringinase flowed into a microchannel reaction system.

In conclusion, naringinase immobilized on graphene was firstly applied for the isoquercitrin production in a microchannel reactor. Graphene exhibited high enzyme adsorption capacity and maximum adsorbed protein loading (approximately 4 g/g of support) was observed. In a batch reactor, the yield of 90.47 ± 2.89% was obtained under the following conditions: a rutin concentration of 0.1 g/L, a protein loading of 4 g/g of support, temperature of 50 °C, and a reaction time of 1 h. In a microchannel reactor, the yield of 92.24 ± 3.26% was obtained under the optimum conditions: a flow rate of 4 *μ*L/min, a rutin concentration of 0.05 g/L, a temperature of 40 °C, and a reaction time of 20 min. Twelve and ten successive cycles of enzymatic reaction were achieved in a batch or microchannel reactor, with relative enzymatic activities all above 85.51 ± 2.76%. Thus, the application of moving and unsinkable graphene sheets immobilized enzyme in a microchannel reactor is highly promising in the production of fine chemicals.

## Methods

### Immobilization of enzyme

A total of 20 mg of graphene nanoparticles were initially treated with 4 mL of a phosphate buffer (the disodium hydrogen phosphate-citrate buffer pH 7) containing 80 mg of commercially available naringinase. This suspension was then placed in an incubator shaker for 3 h at 50 °C, 180 rpm to obtain effective immobilization. Periodically, samples of the supernatant liquid were withdrawn and protein concentration was analyzed according to the Bradford assay and enzyme activity (triiodide assay). Thereafter, the mixtures were separated in a refrigerated high-speed centrifuge for 10 min at 4 °C, 5000 rpm and carefully washed with phosphate buffer (2 to 3 times) to remove residual free enzyme. The obtained product was stored at 4 to 6 °C prior to use.

### Characterization analysis

The prepared samples of immobilized enzymes were characterized by XRD patterns on a SHIMADZU XRD-6000X diffractometer system. FT-IR spectra were recorded on a BRUKER TENSOR 27. The morphology of graphene-immobilized enzyme was studied by SEM (HITACHI S-4700). The morphology of the immobilized enzyme flowing in the microchannel reactor was studied using a fluorescence inversion microscope system (Nikon ECLIPSE TS100).

### Analysis of rutin and isoquercitrin by HPLC

HPLC quantitative analyses were performed using an H&E Pump P3000A (H&E Co., Ltd., Beijing, China) with a UV–VIS detector (PLC-2, Biochem. Jinda, Ltd., Shanghai, China). The separation and determination of rutin and isoquercitrin using an HPLC-UV method were performed on an Alltima C_18_ column (250 mm × 4.6 mm, i. d.; 5 µm, W. R. Grace & Co., Deerfield, IL, USA) using a mobile phase consisting of an acetonitrile: 0.02% phosphoric acid solution (20:80, v/v) at a flow rate of 1.0 mL/min. Rutin and isoquercitrin were detected at 360 nm. All solutions were filtered through a 0.45 *μ*m filter before injection. The yield of isoquercitrin was calculated using previously reported method^43^.

### Equilibrium isotherm and kinetics of adsorption experiments

Adsorption isotherm experiments are required for the protein determination of immobilized enzyme. The determination of protein was performed by Bradford’s method^49^. Bovine serum albumin (BSA) was prepared as a standard protein in the experiment. In this study, two different adsorption isotherms, Langmuir and Freundlich isotherm models, were used to fit the experimental data obtained from naringinase adsorption on graphene nanoparticles^33^.

Where *q*_*e*_ is the adsorption capacity at equilibrium (g protein per g support), *C*_*e*_ is defined as the residual maximum amount of protein per unit volume of naringinase solution (mg protein/mL), *q*_max_ is the maximum adsorption capacity of naringinase (g protein per g support), and *K*_L_ is the Langmuir constant (mL/mg protein).

1 $${q}_{e}=\frac{{q}_{{\rm{\max }}}\times {K}_{L}C{e}^{n}}{1+{K}_{L}C{e}^{n}}$$

Where *K*_F_ is the Freundlich isotherm constant (mL/mg support), and *n* is the Freundlich exponent.

2 $$qe={K}_{F}\times C{e}^{1/n}$$

The adsorption kinetics for enzymes were investigated by pseudo-first-order (3) and pseudo second-order (4) models.

3 $$\mathrm{ln}({q}_{e}-{q}_{t})=\,\mathrm{ln}\,{q}_{e}-{K}_{1}t$$

Where *q*_*e*_ and *q*_*t*_ are the amounts adsorbed in g/g at equilibrium and time ‘*t*’ in min, and *K*_1_ the equilibrium rate constant of pseudo-first-order (min^−1^).

4 $$\frac{t}{{q}_{t}}=\frac{1}{{K}_{2}{{q}_{e}}^{2}}+\frac{t}{{q}_{e}}$$

Where *q*_*e*_ and *q*_*t*_ are the amounts adsorbed in mg/g at equilibrium and time ‘*t*’ in min, and *K*_2_ is the pseudo-second-order rate of adsorption (min^−1^).

### Enzymatic synthesis of isoquercitrin in a batch reactor and a microchannel reactor

Rutin dissolved in a phosphate buffer solution was added to a 10-mL centrifuge tube. Then, the reaction was then performed in an incubator shaker by adding immobilized enzyme to the centrifuge tube. The mixture was shaken at 180 rpm and sampled at 20, 40, 60, 80 and 120 min. The crude hydrolysis products of rutin diluted with methanol were filtered through a 0.45 *µ*m filter prior injection into HPLC. The immobilized enzyme used was prepared using a protein loading of 4 g/g of support; the effects of substrate concentration using different rutin concentration (0.01–0.2 g/L), and reaction temperatures (40–60 °C) on isoquercitrin yield were investigated.

Graphene-immobilized naringinase was applied in a microchannel reactor. Several factors were explored in the enzymatic synthesis of isoquercitrin. Considering the higher pressure achieved by increasing the flow rate and microchannel crushed by immobilized enzyme, surfactants were used to modify immobilized enzyme, rendering them more hydrophilic. In this study, five types of surfactant, including vinyl alcohol polymer (PVA), cetyltrimethylammonium bromide (CTAB), N,N-dimethyl dodecyl amine-N-oxide (DMAO), sodium dodecyl benzene sulfonate (SDBS) and sodium dodecyl sulfonate (SDS), were added to the mixture of immobilized enzyme to create stable dispersions of the immobilized enzyme. Then, different flow rates (2–20 *µ*L/min) and reaction temperatures (30–55 °C) were investigated, respectively.

### Measurements of kinetic and Michaelis constant

The kinetics of immobilized enzyme in a batch reactor can be described by a Michaelis-Menten model^50^. The experimental data are fitted to a Lineweaver–Burk relationship (5), where *V*_max_ is the maximum velocity of the reaction, *K*_m_ is the kinetic constant, and *S* is the substrate concentration. The values for *K*_m_ and *V*_max_ were obtained from the experimental data.

5 $$\frac{1}{V}=\frac{{K}_{m}}{{V}_{{\rm{\max }}}}\frac{1}{S}+\frac{1}{{V}_{max}}$$

Enzyme kinetics in a microchannel reactor was evaluated using the Lilly-Hornby model (6), which was developed for a continuous-flow microfluidic system^51^.

6 $$f[{A}_{0}]=\frac{C}{Q}+{K}_{m(app)}\,\mathrm{ln}(1-f)$$

Where *f* is the fraction of substrate converted to product during the reaction, *Q* is the flow rate of the substrate, [*A*_0_] is the initial substrate concentration, *C* is the reaction capacity of the microreactor, and *K*_m(app)_ is the kinetic parameter.

### Reusability tests of immobilized enzyme

Reusability tests for immobilized enzymes were performed under optimal experimental conditions as follows: in a batch reactor, with previously prepared graphene-immobilized naringinase, a rutin concentration of 0.1 g/L, a reaction temperature of 50 °C and a reaction time of 1 h; in a microreactor, with previously prepared graphene-immobilized naringinase, a sodium dodecyl sulfonate concentration of 1.0 g/L, a rutin concentration of 0.05 g/L, a reaction temperature of 40 °C, and a reaction time of 20 min. At the end of each reaction, the mixture was separated in a refrigerated high-speed centrifuge for 10 min at 4 °C and 5000 rpm and carefully washed with a phosphate buffer (2 to 3 times) to remove residual mixture. Then, a fresh reaction mixture was added to the reaction system.

### Statistical analysis

Triplicate experiments were performed for each parameter investigated. Standard deviations were calculated to verify the reliability of the results. The differences in mean values were evaluated using the analysis of variance (ANOVA). Significance was determined at a 95% level of probability.

## Electronic supplementary material

Supplemental materials
