# Supplementary material for: Moving and unsinkable graphene sheets immobilized enzyme for microfluidic biocatalysis
Source: Sci Rep. 2017 Jun 27;7:4309. doi: 10.1038/s41598-017-04216-4 (PMC5487366; doi:10.1038/s41598-017-04216-4)
Supplement: Supplementary file 1 — Supplemental materials [file 41598_2017_4216_MOESM1_ESM.doc]

**Supplemental materials for**

**Moving and unsinkable graphene sheets immobilized enzyme for microfluidic biocatalysis**

An Gong, Chang-Tong Zhu, Yan Xu, Fang-Qin Wang, D’assise Kinfack Tsabing, Fu-An Wu, Jun Wang

*Adsorption experiments for the immobilization of enzyme on to graphene*

The effects of substrate concentration using different enzyme concentrations (1-25 g/L), PH values, reaction times (1-5h) and reaction temperatures (40-60 °C) on the adsorption experiments of enzyme were investigated. Enzyme powder was dissolved by disodium hydrogen phosphate-citrate buffer; the graphene mass was 10 mg; reaction system was 2 mL; and the mixture was stirred at 120 rpm in an incubator shaker. Thereafter, the mixtures were separated in a refrigerated high-speed centrifuge for 10 min at 4 °C, 5000 rpm and the absorbance of upper residual enzyme solution was determined according to the Bradford´s method. The lower absorbance of upper residual enzyme solution suggests that more enzyme molecules were adsorbed onto surface of the graphene. All experiments were carried out in triplicate.

Figureure. S1A shows that the absorbance was the lowest with the PH value increasing to 7. Figureure. S1B shows the reaction time for enzyme adsorbed onto the surface of graphene, and the optimum reaction time was 3 h. The effect of temperature on the adsorption process was described in Figure. S1C. At reaction temperature of 50 °C, the lowest absorbance of upper residual enzyme solution was achieved. Upon further increase in the reaction temperature, ranging from 50 °C to 65°C, the absorbance showed a significant increase. As shown in Figure. S1D, Different concentrations of enzyme solution were used for the adsorption experiments. With an increase of enzyme concentrations, the absorbance of the supernatant increased. However, by the addition of enzyme concentration reaching 20 g/L, no significant increase in the absorbance of the supernatant was observed. With the enzyme concentration of 20 g/L, the immobilized protein loading (1.13 g/g) and specific activity (234.93 U/g_IP_) were both highest, though the immobilization yield was lower. Hence, the immobilized protein loading (4 g/g of support) was selected was considered the most efficient and selected for subsequent studies.

Immobilization yield, immobilized protein loading and specific activity were calculated as shown in Eq. (S1, S2 and S3):

$\text{Immobilization yield }\left(\%\right)=\frac{HA_{0}-HA_{f}}{HA_{0}}\times100$ (S1)

*HA_0_* is the hydrolytic activity in the supernatant before immobilization (IU/mL) and *HA_f_* is the hydrolytic activity in the supernatant after immobilization (IU/mL).

$\text{Immobilized protein loading }\left(\text{g/g of support}\right)=\frac{\text{Amount of coupled proteins}}{\text{Amount of nanoparticles}}$ (S2)

$\text{Specific activity (U/g}_{\text{IP}}\text{)}=\frac{\text{Hydrolytic activity of the biocatalysts prepared}}{\text{Immobilized protein loading}}$ (S3)

*Effect of different surfactants on the graphene dispersion* *and isoquercitrin yield*

Five types of surfactant were added in water phase for obtaining the graphene dispersion and maintaining isoquercitrin yield. The surfactants were vinylalcohol polymer (PVA), sodium dodecyl sulfate (SDS), cetyl trimethyl ammonium bromide (CTAB), sodium dodecyl benzene sulfonate (SDBS) and N, N-dimethyldodecylamine-N-oxide (DMAO), respectively. The photo images of graphene dispersions were described in Figure. S2A. The results show that by adding the SDS, SDBS and PVA (0.5 g/L) graphene sheets can more stably disperse in water. Figure. S2B shows effect of five types of surfactant on isoquercitrin yield in a batch reactor; the concentration of surfactants ranges from 0.5 to 3 g/L. By adding the SDS and PVA (0.5 g/L) to the reaction system, the yield of isoquercitrin increased to 86.47 % and 87.75 %, while it reached 85.09 % yield without any surfactant. The result was because that the SDS and PVA could prohibit from the activity loss of enzyme in in aqueous solution. Upon further increase in the concentration of the surfactant, ranging from 1 to 3 g/L, the isoquercitrin yield showed a decrease. However, the isoquercitrin yield with the SDS (1 g/L) can still obtain 84.70 %. Hence, SDS (1 g/L) was selected as the optimum surfactant for further study.

*Characterization*

The morphology of graphene immobilized enzyme was studied by SEM (HITACHI S-4700). MW-CN, MWC-OH and SMW-OH nanoparticles were also used as carriers for the immobilization. The SEM photos were shown in Figure. S3.

*Standard curve of rutin, isoquercitrin and quercetin*

Isoquercitrin with standard products was set for different concentrations to inject to the HPLC inlet for detecting the peak area, repeated each concentration of the standard rutin, isoquercitrin and quercetin for three times and took the average values of the three peak area. Plotting the peak area and the standard corresponding concentration using Origin Pro8, then a linear was fitted with these values and the standard curve of rutin, isoquercitrin and quercetin were obtained, the standard curve of rutin, isoquercitrin and quercetin were shown in Figure. S4.

Regressing rutin, isoquercitrin and quercetin peak area (*Y*) for rutin (A), isoquercitrin (B) and quercetin (C) concentration (*X*), the regression equation was as follows in Eq. (S4, S4 and S6):

*Y*=22000700*X*+33783, R^2^=0.9997 (S4)

*Y*=22208400*X*+110855, R^2^=0.9995 (S5)

*Y*=74002900*X*-73531, R^2^=0.9997 (S6)

It shows that rutin, isoquercitrin and quercetin content and its peak area have a good linear relationship in the range of their concentration. In the present study, the by-product of quercetin (ca. yield = 2.83±1.06 %) was extremely few synthesized. Therefore, we did not discuss it in the text.

*Determination of protein*

In this study，protein was determined according to the Bradford´s method. Bovine serum albumin (BSA) was used as standard protein. The standard curve of protein was shown in Figure. S5.

Regressing protein absorbance (*Y*) for protein concentration (*X*), the regression equation was as follows in Eq. (S7):

*Y*=0.45763*X*+0.01857，R^2^=0.9996 (S7)

**Supplementary Figure Legends:**

**Figure. S1**. Effects of pH value (A), reaction time (B), reaction temperature (C), and enzyme concentration (D) on the adsorption capacity of graphene. Reaction conditions: the graphene mass was 10 mg; reaction system was 2 mL; and the mixture was stirred at 120 rpm in an incubator shaker. (A) the enzyme powder was dissolved in 20 g/L by disodium hydrogen phosphate-citrate buffer (pH values ranges from 5 to 11); reaction temperature was 50 °C; (B) the mixture was obtained at 1, 2, 3, 4 and 5 h; enzyme powder was dissolved in 20 g/L by disodium hydrogen phosphate-citrate buffer ( pH=7); and reaction temperature was 50 °C; (C) enzyme concentrations were 1, 2, 5, 7.5,10, 12.5, 15, 20, and 25 g/L; the pH value was 7, reaction time was 3 h, and reaction temperature was 50 °C; (D) reaction temperatures were 40, 45, 50, 55 and 60 °C, enzyme concentration was 20 g/L, the pH value was 7, and reaction time was 3 h.

**Figure. S2**. The photoimages of graphene dispersions after adding the same amount of different surfactants in water phase for standing 2 h (A); surfactants concentration 1 g/L; graphene mass 10 mg; graphene can stably disperse in water with surfactants. Effect of five types of surfactant on isoquercitrin yield in a batch reactor (B); the concentration of surfactants ranges from 0.5 to 3.0 g/L.Vinylalcohol polymer (PVA); sodium dodecyl sulfate (SDS); cetyl trimethyl ammonium bromide (CTAB); sodium dodecyl benzene sulfonate (SDBS); N,N-dimethyldodecylamine-N-oxide (DMAO). Reaction conditions: In a batch reactor, rutin concentration was 0.1 g/L, enzyme concentration 50g/L, the reaction time was 8 h and the reaction temperature was 40 °C.

**Figure. S3**. The SEM photos of MW-CN, MWC-OH and SMW-OH nanoparticles immobilized enzyme: (A), (C) and (E) before immobilizing, (B), (D) and (F) after immobilizing. Reaction condition: the temperature is 50 ◦C; the enzyme powder was confected in 20 g/L by 2 mL disodium hydrogen phosphate-citrate buffer (pH 7); the nanoparticles mass was 10 mg; the reaction time was 2 h; the mixture was stirred at 120 rpm in an incubator shaker.

**Figure. S4**. Standard curve plots of rutin (A), isoquercitrin (B) and quercetin (C). (A) The concentrations of rutin ranged from 0.01 to 0.2 g/L, (B) The concentrations of isoquercitrin ranged from 0.05 to 1.0 g/L. (C) The concentrations of ranged from quercetin 0.01 to 0.25 g/L.

**Figure. S5**. Standard curve plots of protein. Protein concentrations ranged from 0.1 to 1.0 g/L.


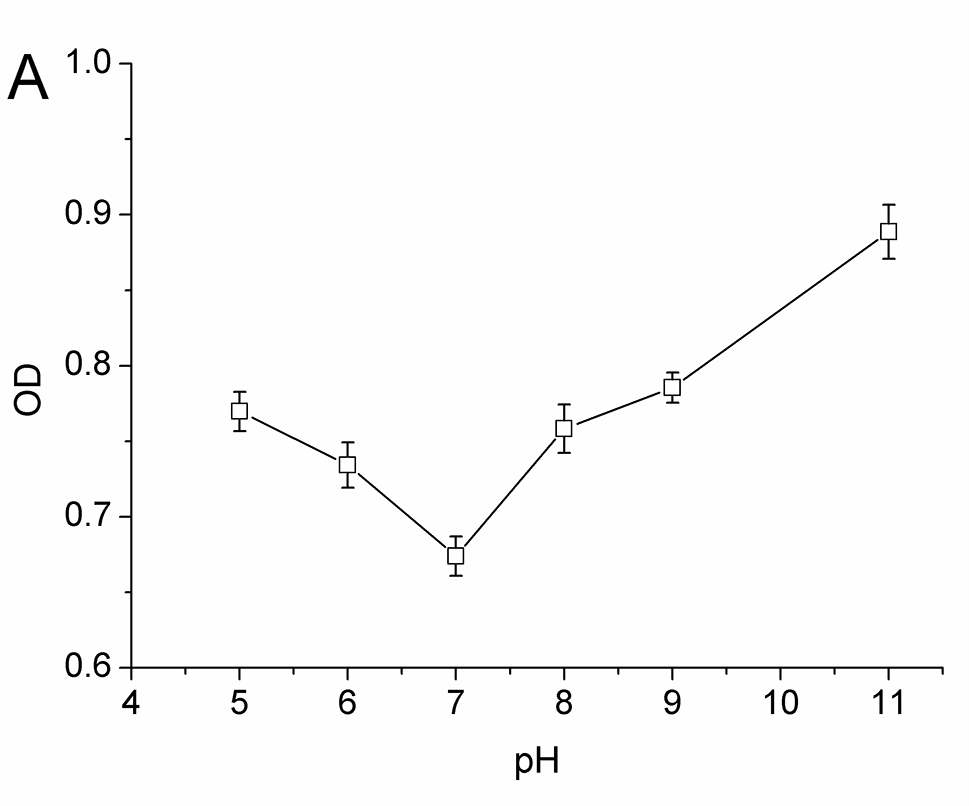

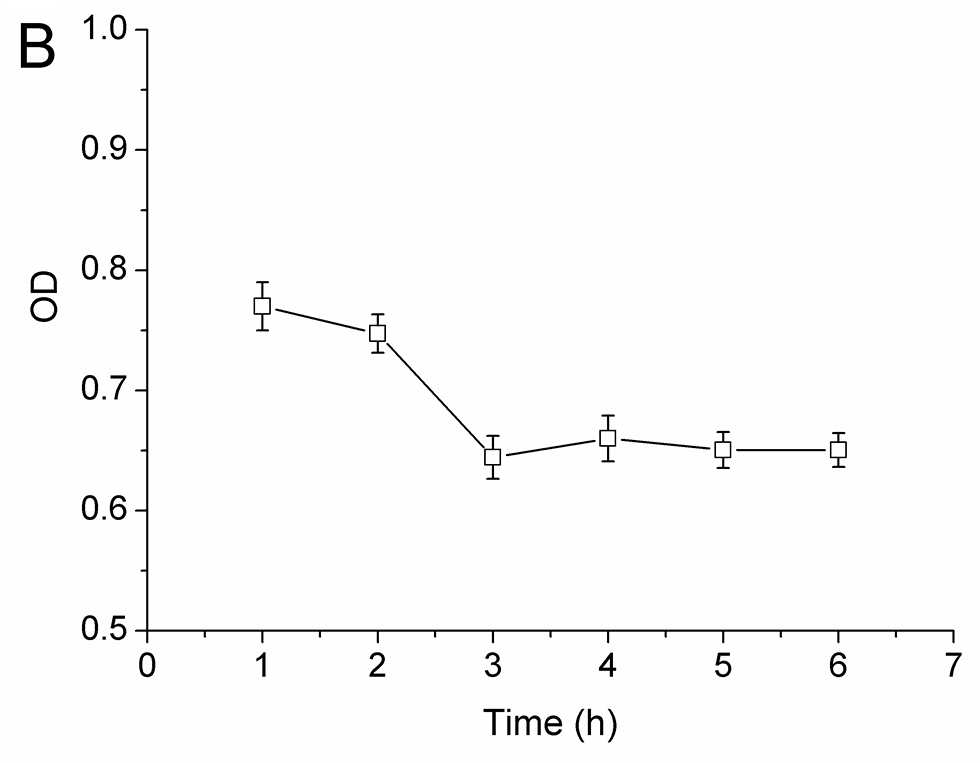


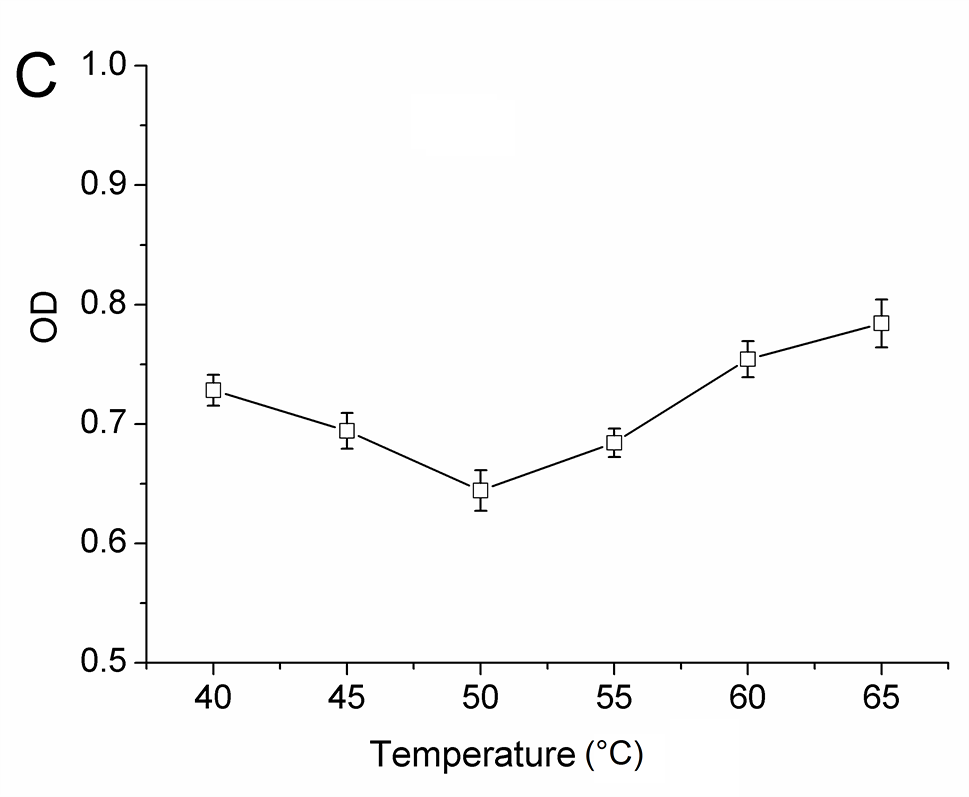

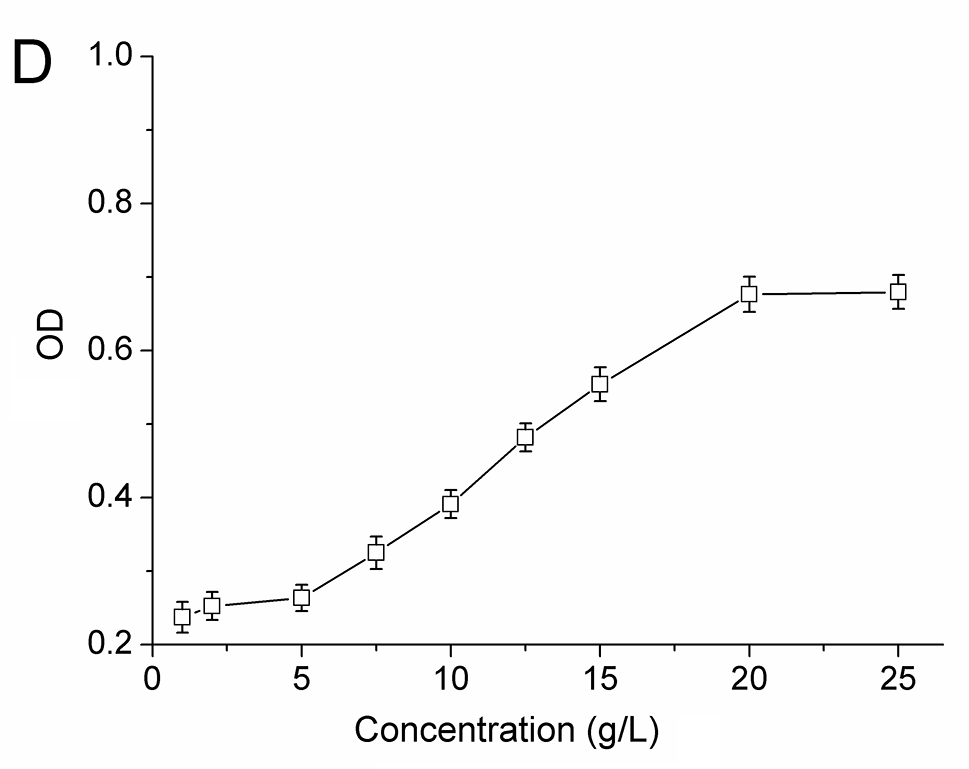


**Figure. S1**. Effects of pH value (A), reaction time (B), reaction temperature (C), and enzyme concentration (D) on the adsorption capacity of graphene. Reaction conditions: the graphene mass was 10 mg; reaction system was 2 mL; and the mixture was stirred at 120 rpm in an incubator shaker. (A) the enzyme powder was dissolved in 20 g/L by disodium hydrogen phosphate-citrate buffer (pH values ranges from 5 to 11); reaction temperature was 50 °C; (B) the mixture was obtained at 1, 2, 3, 4 and 5 h; enzyme powder was dissolved in 20 g/L by disodium hydrogen phosphate-citrate buffer ( pH=7); and reaction temperature was 50 °C; (C) enzyme concentrations were 1, 2, 5, 7.5,10, 12.5, 15, 20, and 25 g/L; the pH value was 7, reaction time was 3 h, and reaction temperature was 50 °C; (D) reaction temperatures were 40, 45, 50, 55 and 60 °C, enzyme concentration was 20 g/L, the pH value was 7, and reaction time was 3 h.


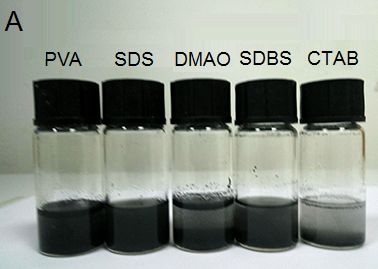


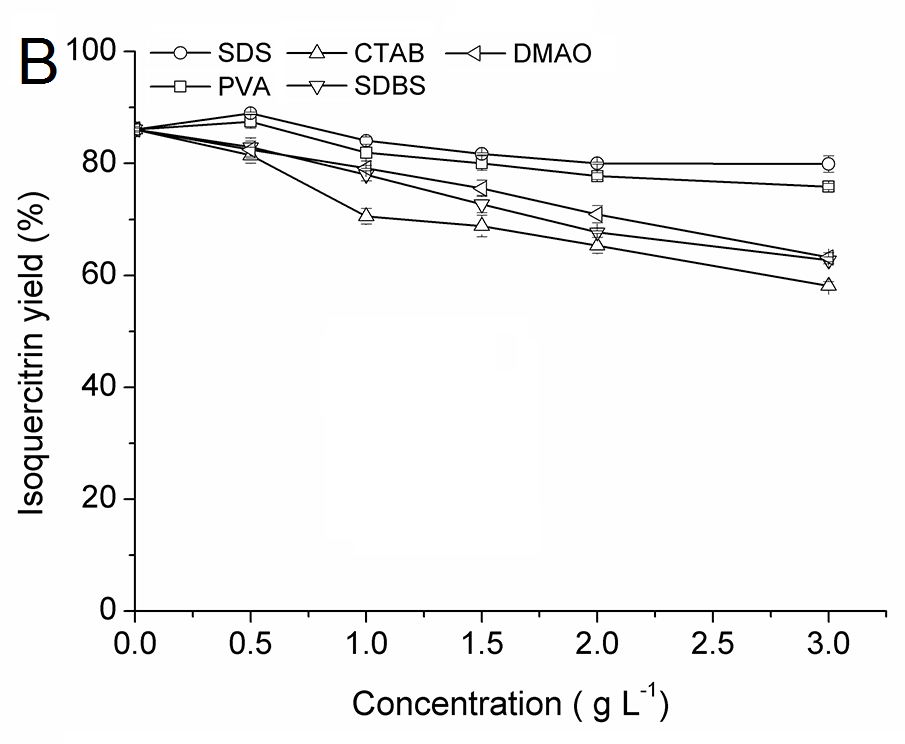


**Figure. S2**. The photoimages of graphene dispersions after adding the same amount of different surfactants in water phase for standing 2 h (A); surfactants concentration 1 g/L; graphene mass 10 mg; graphene can stably disperse in water with surfactants. Effect of five types of surfactant on isoquercitrin yield in a batch reactor (B); the concentration of surfactants ranges from 0.5 to 3.0 g/L.Vinylalcohol polymer (PVA); sodium dodecyl sulfate (SDS); cetyl trimethyl ammonium bromide (CTAB); sodium dodecyl benzene sulfonate (SDBS); N,N-dimethyldodecylamine-N-oxide (DMAO). Reaction conditions: In a batch reactor, rutin concentration was 0.1 g/L, enzyme concentration 50 g/L, the reaction time was 8 h and the reaction temperature was 40 °C.


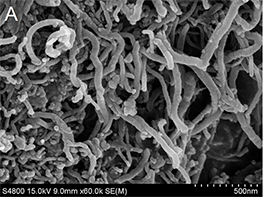

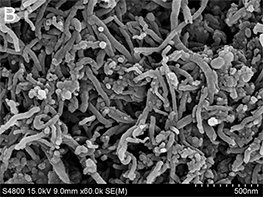

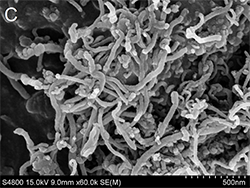

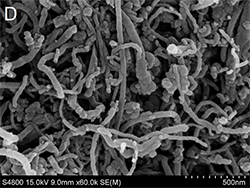

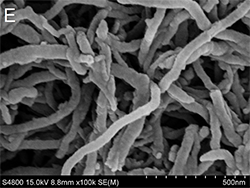

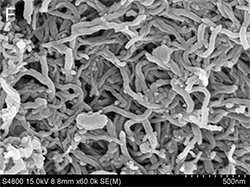


**Figure. S3**. The SEM photos of MW-CN, MWC-OH and SMW-OH nanoparticles immobilized enzyme: (A), (C) and (E) before immobilizing, (B), (D) and (F) after immobilizing. Reaction condition: the temperature is 50 ◦C; the enzyme powder was confected in 20 g/L by 2 mL disodium hydrogen phosphate-citrate buffer (pH 7); the nanoparticles mass was 10 mg; the reaction time was 2 h; the mixture was stirred at 120 rpm in an incubator shaker.


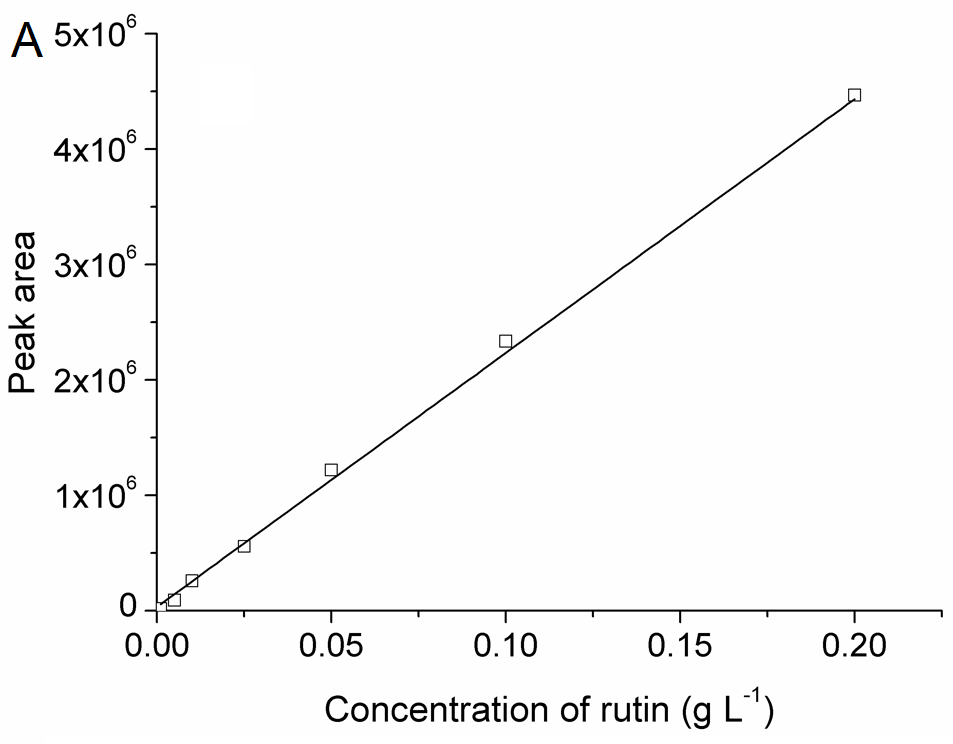


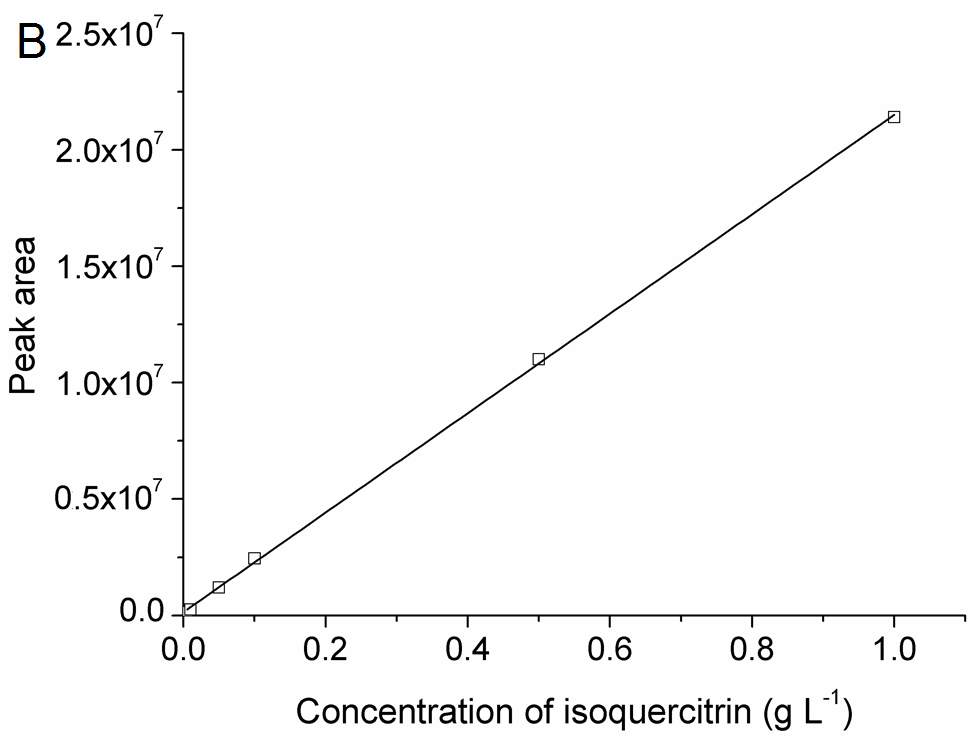


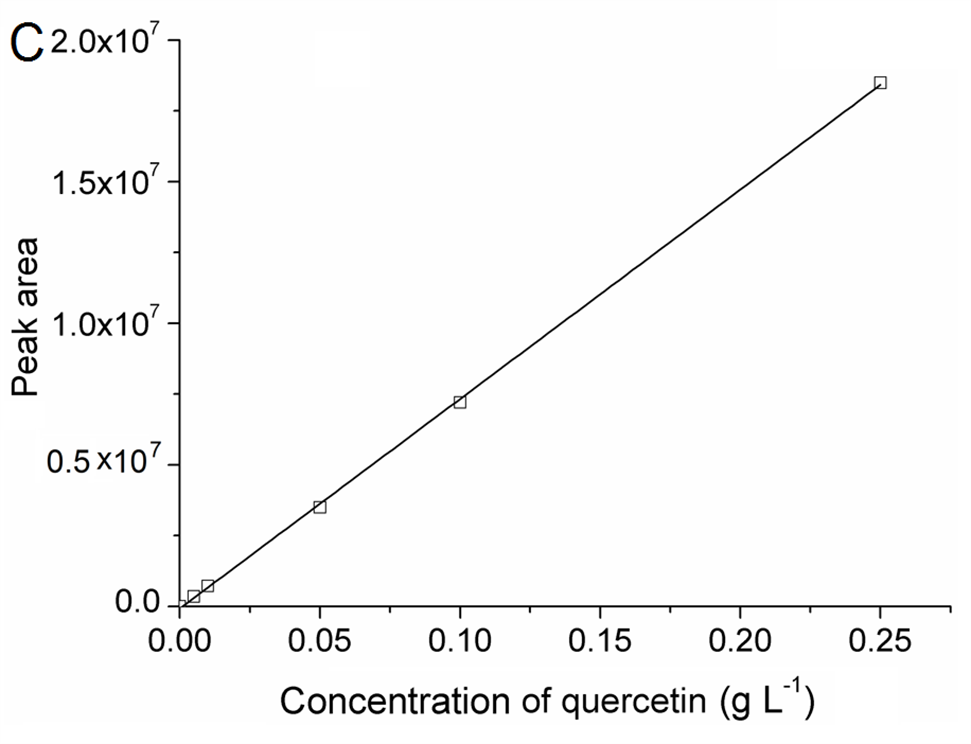


**Figure. S4**. Standard curve plots of rutin (A), isoquercitrin (B) and quercetin (C). (A) The concentrations of rutin ranged from 0.01 to 0.2 g/L, (B) The concentrations of isoquercitrin ranged from 0.05 to 1.0 g/L. (C) The concentrations of ranged from quercetin 0.01 to 0.25 g/L.


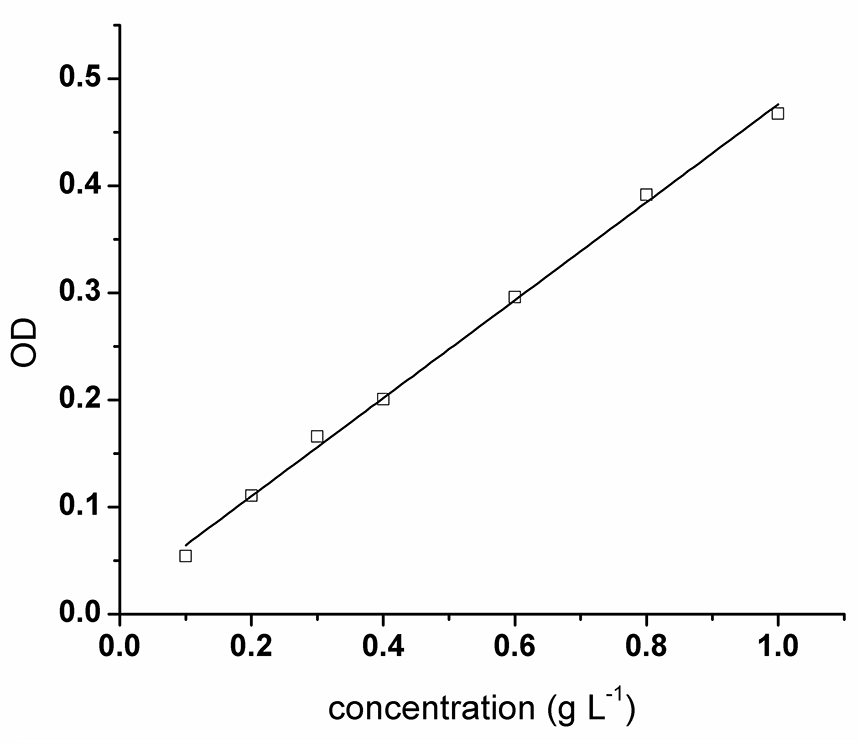


**Figure. S5**. Standard curve plots of protein. Protein concentrations ranged from 0.1 to 1.0 g/L.
